# The *In Vivo* and *In Vitro* Architecture of the Hepatitis C Virus RNA Genome Uncovers Functional RNA Secondary and Tertiary Structures

**DOI:** 10.1128/jvi.01946-21

**Published:** 2022-03-30

**Authors:** Han Wan, Rebecca L. Adams, Brett D. Lindenbach, Anna Marie Pyle

**Affiliations:** a Department of Molecular, Cellular, and Developmental Biology, Yale University, New Haven, Connecticut, USA; b Department of Microbial Pathogenesis, Yale University School of Medicine, New Haven, Connecticut, USA; c Department of Chemistry, Yale University, New Haven, Connecticut, USA; d Howard Hughes Medical Institute, Chevy Chase, Maryland, USA; University of Southern California

**Keywords:** HCV, SHAPE, pseudoknot, RNA folding, viral replication

## Abstract

Hepatitis C virus (HCV) is a positive-strand RNA virus that remains one of the main contributors to chronic liver disease worldwide. Studies over the last 30 years have demonstrated that HCV contains a highly structured RNA genome and many of these structures play essential roles in the HCV life cycle. Despite the importance of riboregulation in this virus, most of the HCV RNA genome remains functionally unstudied. Here, we report a complete secondary structure map of the HCV RNA genome *in vivo*, which was studied in parallel with the secondary structure of the same RNA obtained *in vitro*. Our results show that HCV is folded extensively in the cellular context. By performing comprehensive structural analyses on both *in vivo* data and *in vitro* data, we identify compact and conserved secondary and tertiary structures throughout the genome. Genetic and evolutionary functional analyses demonstrate that many of these elements play important roles in the virus life cycle. In addition to providing a comprehensive map of RNA structures and riboregulatory elements in HCV, this work provides a resource for future studies aimed at identifying therapeutic targets and conducting further mechanistic studies on this important human pathogen.

**IMPORTANCE** HCV has one of the most highly structured RNA genomes studied to date, and it is a valuable model system for studying the role of RNA structure in protein-coding genes. While previous studies have identified individual cases of regulatory RNA structures within the HCV genome, the full-length structure of the HCV genome has not been determined *in vivo*. Here, we present the complete secondary structure map of HCV determined both in cells and from corresponding transcripts generated *in vitro*. In addition to providing a comprehensive atlas of functional secondary structural elements throughout the genomic RNA, we identified a novel set of tertiary interactions and demonstrated their functional importance. In terms of broader implications, the pipeline developed in this study can be applied to other long RNAs, such as long noncoding RNAs. In addition, the RNA structural motifs characterized in this study broaden the repertoire of known riboregulatory elements.

## INTRODUCTION

RNA viruses provide a unique source of information on RNA structure. Because their genomes are relatively compact, RNA viruses must maximize the utility of RNA, using both primary sequence and structure to convey information, often within the same sequence. This “code within code” strategy provides viruses with extra layers of regulation. However, we still have limited understanding of RNA structure-based regulation and its contribution to viral growth and host-pathogen interactions.

Hepatitis C virus (HCV) is a 9.6-kb positive-strand RNA virus in the *Flaviviridae* family (1). With many well-established assays that report on diverse aspects of the viral life cycle, including viral replication, translation, and packaging (2, 3), HCV has become a powerful model system for understanding functional mechanisms in RNA viruses. Previous studies have demonstrated that the HCV genome is rich in complex RNA structures, containing functional RNA motifs in both the untranslated regions (UTRs) and the coding regions (4–10). For example, the HCV internal ribosome entry site (IRES) within the 5′ UTR is among the best-characterized IRES structures in any system (11). The core region of HCV is known to contain highly conserved substructures, of which many are involved in long-range interactions that play important roles in virus replication and translation (5, 12, 13). RNA structures in the NS5B protein-coding region serve as *cis*-acting regulatory elements that are essential for both HCV replication and viral protein synthesis (14, 15). A dynamic stem in the region encoding protein NS4B, termed stem-loop 6038 (SL6038) undergoes conformational changes that regulate replication (5). Finally, a highly conserved RNA motif located in the region encoding protein NS5B, termed J7800 contributes to both replication and infection (6).

Riboregulatory structures, such as knots, pseudoknots and kissing loop interactions, are clearly important in the life cycle of other positive-strand viruses, such as flaviviruses (16), so it is important to evaluate how pervasive these interactions are throughout the HCV genome. In most cases, these elements have been identified in UTRs, and it is therefore of particular interest to determine if they are found in coding regions of HCV and other positive-strand RNA viruses.

Despite many advances, our knowledge of functional RNA motifs has not yet been extended to the full-length HCV genome in a cellular context, particularly in the coding region. Previous studies on molecular architecture within the full-length genome were conducted with RNA that was transcribed with T7 RNA polymerase, refolded, and manipulated *in vitro* (5, 6). While this information has been valuable, it is essential to conduct in-cell RNA probing experiments that will reflect discrete RNA structural states during the course of infection and replication and to reveal potential sites of protein binding.

In this study, we generated a full-length HCV structure map, including pseudoknots, for both *in vitro* purified RNA and *in vivo* extracted RNA using selective 2′-hydroxyl acylation analyzed by primer extension, read out by mutational profiling (SHAPE-MaP). We show that the HCV RNA is highly structured both *in vitro* and *in vivo*, identifying 20 well-folded regions in the HCV genome. Relying on the analysis of synonymous mutation rates (dS), base pair content (BPC), and differential reactivities between the two experimental conditions, we found highly structured motifs with evolutionary support as well as potential protein binding sites. We also identified three novel pseudoknots in the HCV coding region and demonstrated with structure-disrupting and compensatory mutations that all three play important roles in HCV replication. Overall, we present a detailed map of HCV secondary and tertiary structure and show that the viral open reading frame (ORF) contains compact structures that are important for viral growth. The methods of analysis and quality control used in this study can be extended readily to the study of other RNA viruses and other long RNA transcripts.

## RESULTS

### Semi-natively folded HCV RNA yields well-predicted regions of RNA structure.

**FIG 1:**
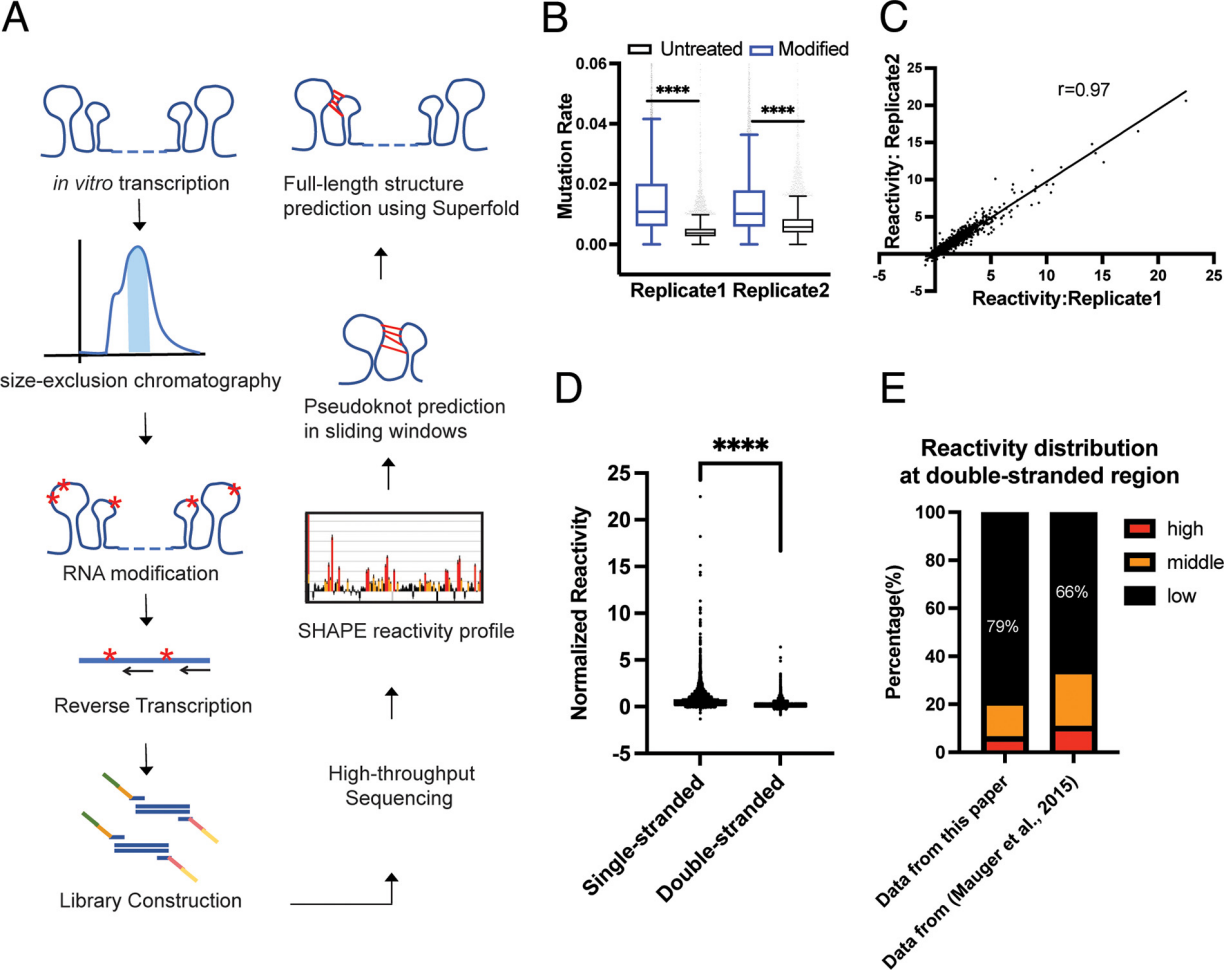
Semi-native folded HCV RNA is of sufficient quality for structure discovery. (A) Workflow of *in vitro* SHAPE-MaP and structure prediction. (B) Mutation rate of two biological replicates across the HCV Jc1 genome. The boxes represent interquartile range, the median is indicated by a line, whiskers are drawn in Tukey style, and values outside this range are not shown. (C) Correlation plot of normalized SHAPE reactivities from two biological replicates. The line represents a linear regression fit to the data. (D) Normalized SHAPE reactivity binned by strandedness, as determined by our consensus structural model. (E) Comparison of the SHAPE reactivity distribution of double-stranded regions in our SHAPE data and data that were collected in Mauger et al. SHAPE data (6). Low, reactivity of <0.4; middle, 0.4 ≤ reactivity < 0.85; high, reactivity of ≥0.85; ****, *P* < 0.0001 by equal variance unpaired Student’s *t* test.

To obtain a high-quality *in vitro* structure model that would be suitable for guiding mutational study and downstream functional analysis, we used a recently optimized semi-native purification method (17) to purify the genotype 2a HCV RNA from *in vitro* transcription. The secondary structure of the purified RNA was then probed by using the SHAPE-MaP strategy (18) with the SHAPE reagent 2-methylnicotinic acid imidazolide (NAI) (19). Reactivity profiles were then obtained after high-throughput sequencing, enabling construction of a whole-genome secondary structural model (Fig. 1A). The SHAPE-MaP data set used for the construction of a secondary structure model had an average effective depth of >100,000 reads per site and effective reactivities covering 97% of the nucleotides. Mutation rate analysis demonstrated that the NAI-treated sample had a significantly higher mutation rate than the untreated sample (Fig. 1B) (*P* < 0.0001), indicating that the HCV RNA was modified successfully. The Pearson’s correlation analysis showed that the results are reproducible among different biological replicates (Fig. 1C).

**FIG 2:**
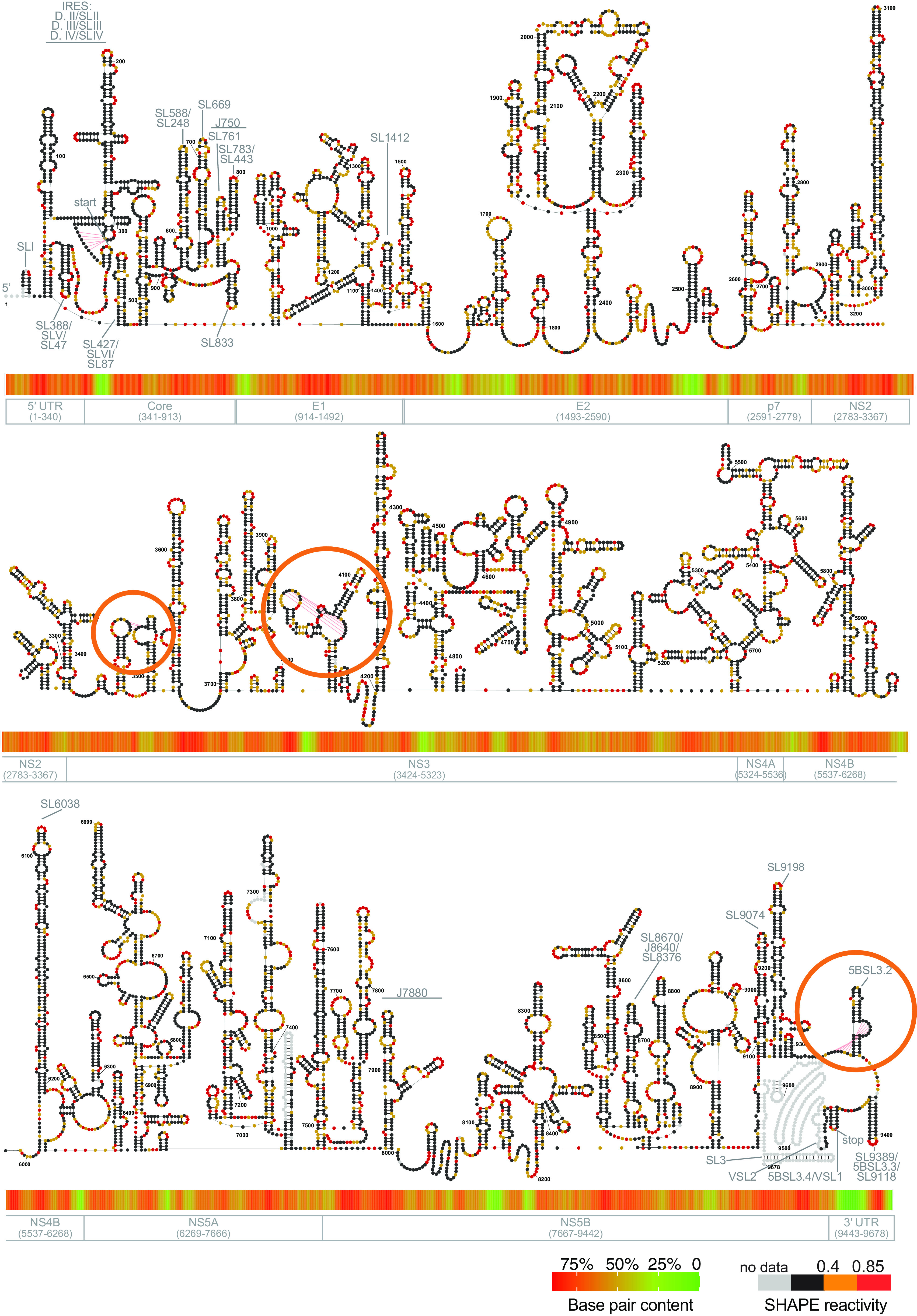
The *in vitro* secondary structural map of HCV. The *in vitro* SHAPE reactivities are color coded, as shown in the legend. A heatmap representing HCV base pair content (BPC) is shown below each section of the structure diagram. The color key to the heatmap is shown on the bottom right corner. Boundaries of HCV coding regions are shown under the heatmap. Gray text (e.g., SL588) indicates regions identified previously. Orange circles indicate the three novel pseudoknots. *In vitro* replicate 1 data were used to generate this structural map.

To build a secondary structure model of the genome from these data, we first performed global pseudoknot prediction by using ShapeKnots (implemented in RNA structure v5.8) (20). Pseudoknots are stable tertiary structures that are identified frequently as important structural elements in viral genomes (reviewed in reference 16). By using the well-characterized HCV IRES pseudoknot as a benchmark (11), we created a set of pseudoknot prediction criteria (Materials and Methods section “pseudoknot prediction”). Using this approach, we then predicted three new pseudoknots across the HCV genome (Fig. 2, orange circles). The experimentally determined SHAPE reactivity was found to be in good agreement with these pseudoknot structures. The full-length HCV secondary structure was then predicted by using Superfold (21) with *in vitro* NAI SHAPE reactivities and the three predicted pseudoknots as constraints.

After we ran both ShapeKnots and Superfold analyses, it was then possible to produce a full-length HCV secondary structural model that includes all high-confidence pseudoknots. To determine how well the experimentally determined SHAPE reactivities agree with the resulting model, we separated our SHAPE reactivities of single-stranded and double-stranded regions that were predicted by our model. The two bins of reactivities separated very well from each other, indicating global agreement of experimental data with the predicted model (Fig. 1D) (*P* < 0.0001). In contrast, when we separated the SHAPE reactivities of single-stranded and double-stranded regions from a previously published HCV genomic structure determined by using denatured and refolded RNA (6), only 66% of nucleotides predicted to lie within double-stranded region had low reactivities (<0.4). By comparison, 79% of the nucleotides that are predicted to be double stranded in our model displayed low reactivities (Fig. 1E). Taken together, these data suggest that the semi-native purification method generates a relatively homogenous, monodisperse population of RNA, resulting in a structural model that agrees well with measured SHAPE reactivities.

Implementation of the secondary structural modeling pipeline described above resulted in a well-predicted secondary structural map that was highly consistent among different biological replicates (87.37% base pair similarity) (Fig. 2). Inspection of the secondary structural map revealed that the viral genome is highly structured, with 60% of nucleotides involved in base pairs. A local base pair content (BPC) analysis (22) showed that different regions of HCV RNA have various levels of secondary structure content (Materials and Methods section “Local base pair content (BPC) analysis”). For example, sections of the RNA encoding E2, NS3, and NS5B were less structured than other regions in the genome (Fig. 2). In addition, we found that most previously determined RNA structures were correctly predicted by our structural model, including the IRES structure, the 3′ X structure in the 3′ UTR, the *cis* regulating element (CRE) in the NS5B, and the long-stem structure in NS4B(SL6038) (reviewed in reference 4 and labeled in Fig. 2), which further supports the high quality of this *in vitro* structure model. Importantly, we noticed that there were numerous long-stemmed structures within the genomic RNA, such as SL3020 in NS2, SL5724 in NS4B, SL7030 in NS5A, and SL8431 and SL9074 in NS5B. The median base pair distance calculated for HCV was 38 nucleotides (nt), which is higher than that reported for the SARS-CoV-2 genome (25 nt, median; *P* < 0.0001) (23) and dengue virus (33 nt, median) (24), indicating that HCV contains a large fraction of long-range base-pairing interactions.

**FIG 3:**
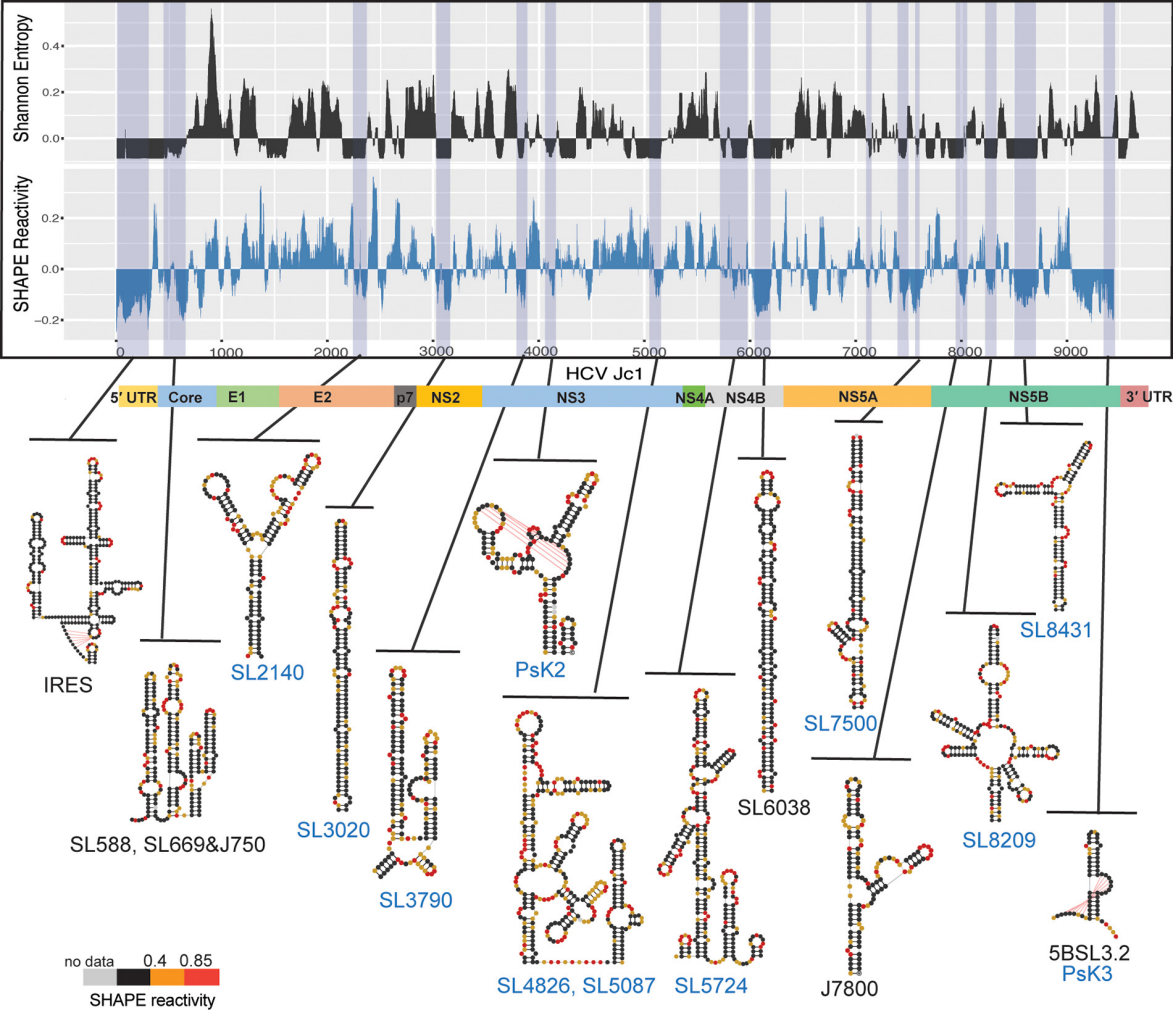
Well-folded HCV secondary structures were determined from the *in vitro* SHAPE-MaP analysis. The local median Shannon entropy (50-nt sliding window) and local median SHAPE reactivity (50-nt sliding window) are plotted along the HCV genome (graph at top). Well-folded regions are shaded in blue. A selection of specific, representative structures is shown at the bottom and labeled with *in vitro* SHAPE reactivities. Previously characterized structures are labeled in black, while novel substructures are named according to their position and labeled in blue.

In order to determine the most compact, well-determined regions of the genome, we computed local median Shannon entropy values and plotted them, together with SHAPE reactivities, at each position along the HCV genome, thereby enabling us to correlate regions of low SHAPE reactivity with low Shannon entropy (Fig. 3). Shannon entropy was determined from base pair probability calculations performed during the Superfold structure prediction (21). Low Shannon entropy suggested strong base-pairing probability for a single RNA structure and limited sampling of alternative substructures (25). Regions with Shannon entropy and SHAPE reactivity values that fell below the global median for ≥40 continuous nucleotides were considered to be “well-folded” regions that adopt discrete structures (Materials and Methods section “Identification of well-folded regions”). In total, we identified 18 well-folded regions in the HCV genome using *in vitro* data; representative structural motifs are shown in Fig. 3 and Table 1. Many of these elements are completely novel, thereby setting the stage for a future functional analysis on their role in the viral life cycle. The RNA substructures that we discovered were named according to their position on the chimeric HCV genome Jc1 (Fig. 3, novel structures in blue). Initial insights into their roles were provided by subsequent *in vivo* probing studies conducted during the second phase of the study (see below).

**TABLE 1:** Well-folded structures characterized using *in vivo* and *in vitro* SHAPE-MaP

Index	RNA structural elements	Sequence interval (HCV-Jc1)	Genome region	Notes^*a*^
1	IRES	1–350	5′ UTR	Both
2	Core regions	386–750	Core	*In vitro*
3	SL2140	2140–2282	E2	*In vitro*
4	SL3020	3020–3160	NS2	*In vitro*
5	SL3790	3790–3941	NS3	Both
6	PsK2	4009–4149	NS3	Both
7	SL4826	4826–5075	NS3	Both
8	SL5087	5087–5159	NS3	Both
9	SL5724	5724–5996	NS4B	Both
10	SL6038	6038-6186	NS4B	Both
11	SL7030	7030–7212	NS5A	Both
12	SL7424	7424–7482	NS5A	Both
13	SL7500	7500–7659	NS5A	Both
14	J7800	7880–7998	NS5B	*In vitro*
15	SL8209	8209–8412	NS5B	*In vitro*
16	SL8431	8431–8644	NS5B	Both
17	SL9074	9103–9166	NS5B	*In vivo*
18	SL9198	9198–9257	NS5B	*In vivo*
19	PsK3/5BSL3.2	9321–9380	NS5B	Both
20	5BSL3.3	9389–9419	NS5B	Both

a Whether the structure is characterized as well-folded using *in vivo* data, *in vitro* data, or both.

### *In vivo* SHAPE analysis showed that HCV is highly structured in the cellular context.

Despite the high quality of the *in vitro* structural map, it was important to obtain a parallel map that captured conformational states of the HCV genome in a cellular context. A comparison of the two maps provided information on highly stable secondary structures, and it also flagged potential protein binding sites and revealed RNA elements that sample multiple functional conformations.

**FIG 4:**
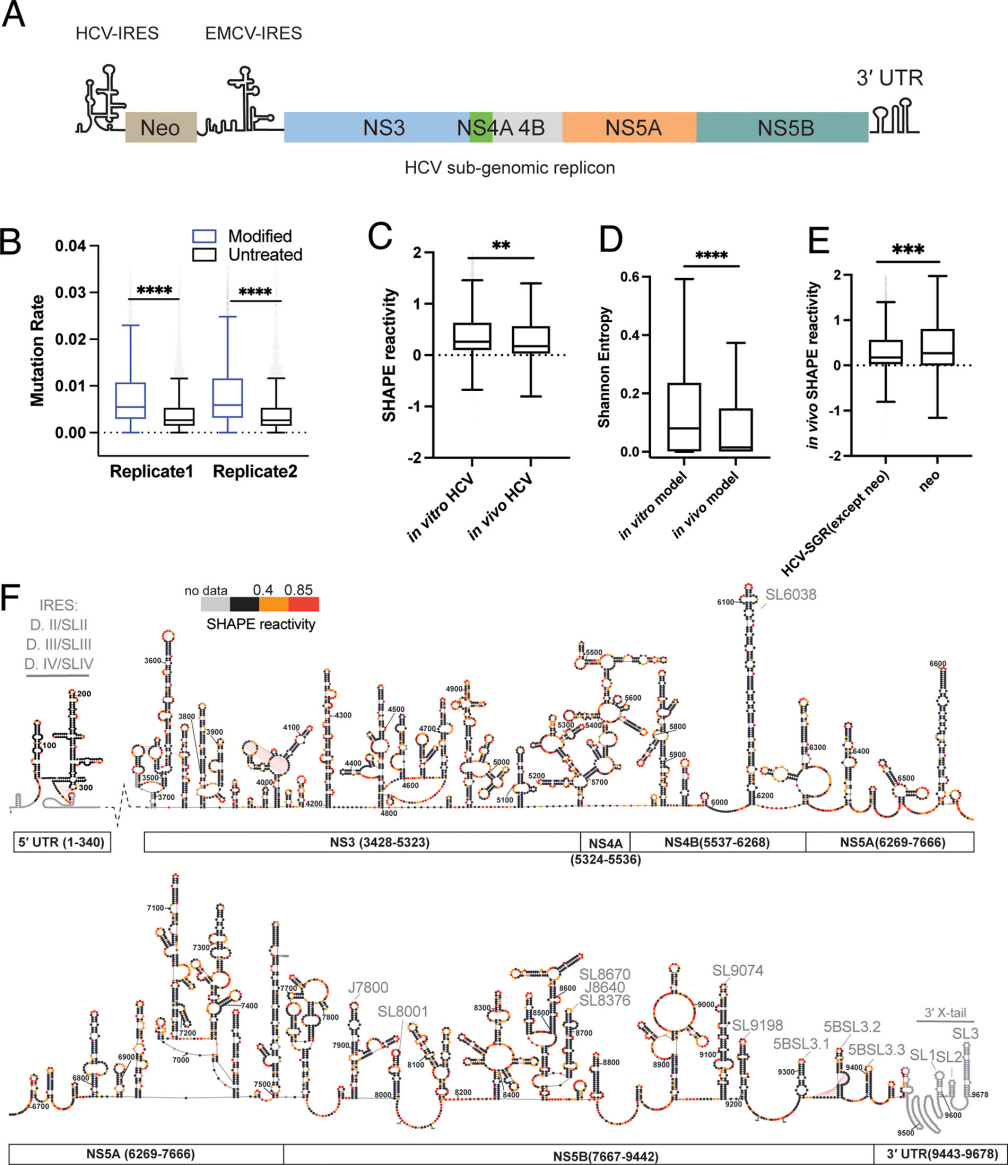
The HCV genome is highly structured in a cellular context. (A) Schematic of the HCV subgenomic replicon (SGR), with constituent genes indicated by colored bars. (B) The mutation rate of two biological replicates across the HCV SGR genome. (C) Normalized SHAPE reactivity distribution of *in vitro* and *in vivo* purified HCV RNA for region NS3-3′ UTR. (D) Shannon Entropy distribution between the *in vitro* and *in vivo* structural models for the NS3-3′ UTR region. (E) The *in vivo* SHAPE reactivity distribution between the HCV-SGR (except neomycin region) and the neomycin region. For panels B to E, data are plotted as in Fig. 1B. (F) The *in vivo* structure model of HCV SGR labeled by SHAPE reactivity. Nucleotides were numbered according to their position on the full-length HCV genome (Jc1). Schematic of corresponding HCV domains is shown below the structural map. Gray text (e.g., IRES) indicates a region previously identified. *In vivo* replicate1 data were used to generate this structure map. The neomycin resistance gene and EMCV IRES were not included in the structure prediction. The full-length structural model, including neomycin resistance gene and EMCV IRES, can be found in the GitHub repository. **, *P* < 0.01; ***, *P* < 0.001; ****, *P* < 0.0001 by equal variance unpaired Student’s *t* test.

To create an *in vivo* SHAPE-MaP library, we used the well-established HCV subgenomic replicon (HCV-SGR) cell culture model (26). This construct contains a neomycin resistance gene (neo) driven by the HCV IRES and HCV nonstructural proteins (NS3-NS5B) driven by the encephalomyocarditis virus (EMCV) IRES (Fig. 4A). Huh-7.5 cells that constitutively support HCV-SGR replication were treated with SHAPE reagent NAI or a dimethyl sulfoxide (DMSO) control solution. Following treatment with the chemical probe, cellular RNA was then extracted and purified for sequencing. The HCV-SGR can be fully amplified by 15 overlapping 700-nt amplicons, which were individually reverse transcribed and PCR amplified. Two biological replicates of the *in vivo* SHAPE-MaP library were generated and comprehensive data sets were obtained, with an average effective depth of ≥200,000 reads per site and effective SHAPE reactivity covering 98.7% of the HCV-SGR genome. Mutation rate analysis showed that for both replicates the HCV RNA was modified successfully (Fig. 4B).

To determine whether the *in vivo* SHAPE data were of sufficient quality for *de novo* structure prediction, we first tested the structure prediction accuracy at the IRES region. We found both replicates predicted the IRES at 95% to 100% accuracy with low overall Shannon entropy (average Shannon entropy, 0.017), thereby demonstrating that the data were suitable for *de novo* structure prediction.

We then set out to predict the remaining genomic structural features by using the *in vivo* SHAPE data set. Since the *in vivo* reactivity data matched well with structures of the three pseudoknots predicted earlier using the *in vitro* data (not shown), the same pseudoknots were used as hard constraints during subsequent *in vivo* RNA structure prediction, which was conducted by Superfold as described in Materials and Methods. The resulting *in vivo* structural model of the HCV-SGR demonstrated that almost all previously known functional RNA structures in the UTRs and ORF were correctly predicted and that they are stable elements that exist in a cellular context (e.g., IRES, CRE in NS5B, SL6038, J7800, and SL9074 labeled in Fig. 4F). It is significant that we were able to obtain a comprehensive genomic structural map by exclusively using *in vivo* SHAPE reactivity data. While the success of this approach may be attributable to a high abundance of the HCV RNA during replication, and potentially other factors, its underscores the advantages of viral model systems for studying *in vivo* RNA structure.

Because *in vitro* and *in vivo* models provide structural information in two very different contexts, one can compare them to obtain functional insights into the genome. For example, we compared the relative secondary structural content of both models. The base pair content (BPC) analysis showed that both models have 60% BPC, indicating a similar level and type of secondary structure complexity in both cases. Since SHAPE reactivity directly reflects nucleotide flexibility, we then analyzed the reactivity distribution of the two data sets. As shown in Fig. 4C, the relative *in vivo* SHAPE reactivity and the *in vitro* reactivity values fell within a similar range. However, the *in vivo* reactivity was slightly lower than that measured *in vitro* (*P* = 0.0013), suggesting that, despite comparable BPC, the RNA *in vivo* was somewhat less flexible than the RNA *in vitro*. As a second comparison, we compared the Shannon entropy values obtained from both *in vivo* and *in vitro* structural models and found that Shannon entropy values for the *in vivo* model were significantly lower than those for the *in vitro* model (Fig. 4D) (*P* < 0.0001). Since a lower Shannon entropy value indicates that a smaller set of conformations are compatible with the measured SHAPE reactivities (27), these results are consistent with the notion that the RNA is less flexible *in vivo* than *in vitro*, which may be attributable to the crowded cellular environment and/or the effects of RNA-binding proteins on RNA structure.

This observation is in contradiction with what has been reported previously in studies of cellular RNA structure, which suggest that cellular RNA is highly unstructured due to active translational events (28–31). To determine whether high levels of *in vivo* structure are a feature of the HCV genome itself or of the cellular context, we compared the SHAPE reactivity distribution between HCV-SGR and a nonviral gene (neomycin resistance gene) that was cloned within the HCV-SGR RNA. The expression of the neomycin resistance gene and the HCV genes are regulated by the HCV IRES and EMCV IRES, respectively, and both type of IRES drive translation with comparable efficiency (32). Although the neomycin resistance gene and HCV genes are located side-by-side within the same transcript, we observed that that the neomycin cassette displayed significantly higher SHAPE reactivity than the HCV RNA. In addition, the neomycin SHAPE reactivity values were distributed across a large span, having median and 75% percentiles that were two times higher than that of HCV (Fig. 4E). These data suggest that, while both regions are being actively translated *in vivo*, the neomycin RNA is largely unfolded while HCV maintains a highly structured conformation. This finding indicates that the high level of secondary structure we observed is a specific feature of the HCV genome, it is preserved in a cellular context, and it is unaffected by active translation.

### Comparison of SHAPE data sets revealed consistently folded structures and a potential protein binding site.

**FIG 5:**
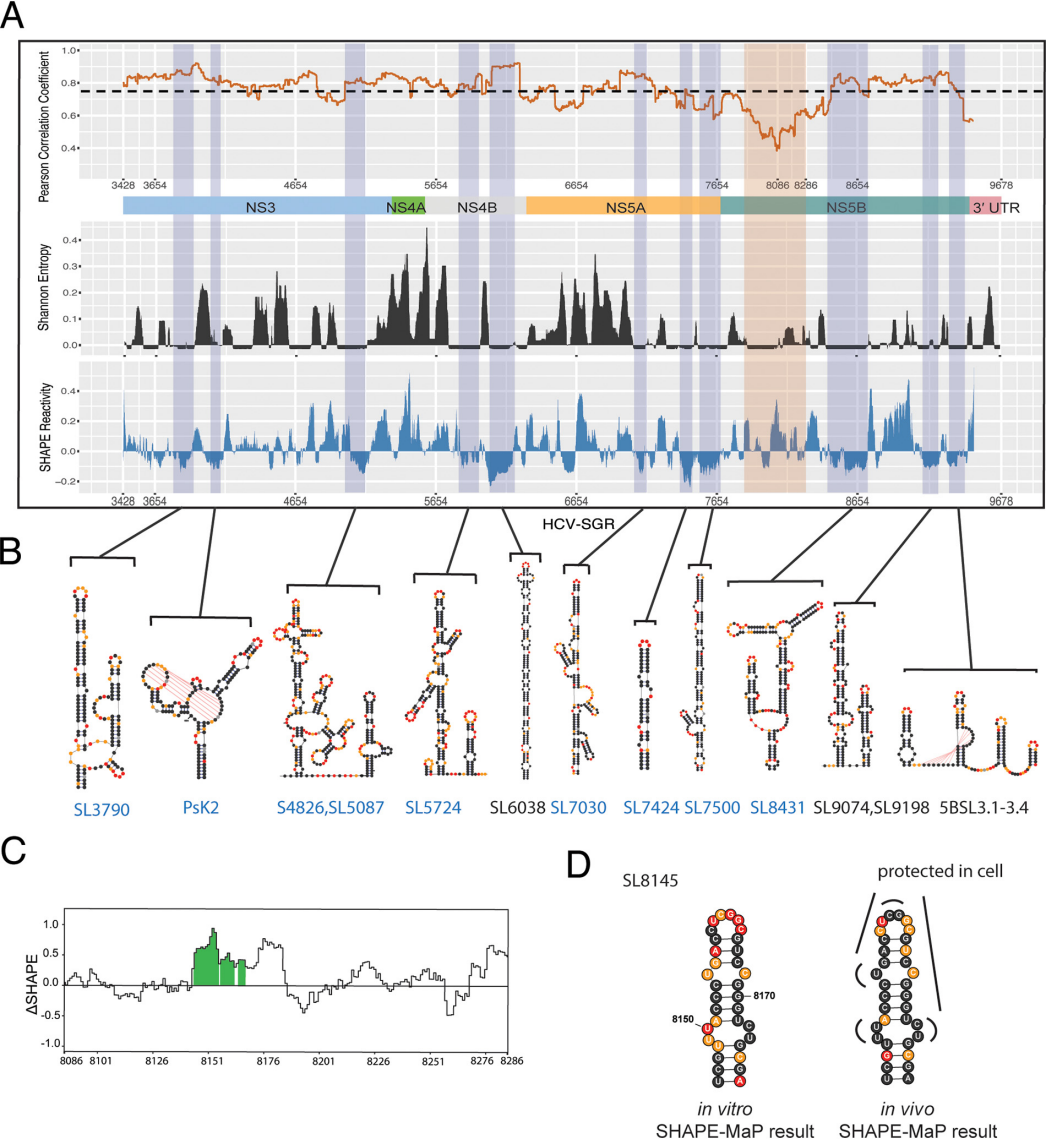
Comparison of *in vivo* and *in vitro* SHAPE data sets (A) Top: The local correlation between *in vitro* SHAPE reactivity and *in vivo* SHAPE reactivity (200-nt sliding window, dashed line represents Pearson *r* = 0.75) as a function of genomic position. A schematic of HCV domains is shown below the graph. Middle and bottom panel: The local median Shannon entropy (50-nt sliding window) and local median SHAPE reactivity (50-nt sliding window) along the HCV SGR genome. Well-folded regions are shaded in blue. A low correlation region (*r* < 0.75, from nucleotides 7877 to 8412 in the HCV genome) is shaded in orange. (B) Structural models for each well-folded region, labeled as in Fig. 3. (C) ΔSHAPE analysis comparing *in vivo* and *in vitro* SHAPE reactivities for nucleotides 8086 to 8286. Regions with significant differences are shaded in green. (D) Structural drawing of SL8145, labeled with *in vitro* SHAPE reactivity (left) and *in vivo* SHAPE reactivity (right).

To further understand the major differences between *in vivo* and *in vitro* SHAPE results, we performed a local correlation analysis of SHAPE reactivities. The local median Shannon entropy and local median SHAPE values were plotted to reveal well-folded regions (Fig. 5A, well-folded regions shaded in blue). A local correlation analysis revealed that the two data sets were highly consistent for 78% of the HCV-SGR genome (Pearson correlation coefficient [r] of >0.75, *r* = 0.75 indicated by a black line in Fig. 5A, top). This section of the genome included the region that encodes NS3-NS5A and the downstream section of NS5B. The predicted secondary structures in these regions were very similar, with 85% consistency in base-pair prediction. In this particular region of the genome, the RNA was much more highly structured relative to the rest of the viral genome. We found that 13 well-folded regions were predicted to be the same in both *in vitro* and *in vivo* models (structures were given the same name according to their position on the full-length HCV genome). They included a bulged stem in NS3 (SL4826), two branched stems in NS4B (SL5724), and NS5B (SL8431) (Fig. 5B, Table 1). These same structures were subsequently shown to be conserved in genotype 2 (see below). In addition, there were two regions (SL9074 and SL9198, Table 1) that were identified as well-folded only by using *in vivo* data and two regions (J7800 and SL8209) (Table 1) that were identified as well-folded only by using *in vitro* data. Even though these structures did not qualify as well-folded regions in both contexts, they still had an almost identical base-pair connectivity between the *in vivo* and *in vitro* structural motifs. Among these regions, SL9074 and SL9198 were identified previously as functional structures in HCV (14, 33). The fact that they were better determined *in vivo* suggested that they are stable elements that exist in the cellular context and highlighted again the high quality of the *in vivo* structure probing data. On the other hand, J7800 and SL8209 were better determined *in vitro*, and they were located in a region of low SHAPE reactivity correlation between *in vitro* and *in vivo*. The significance of this region will be discussed below.

The local correlation analysis also revealed regions of very low correlation (*r* of ∼0.5) within a section encoding domains of NS5B (7877 nt to 8412 nt) (orange-shaded region, Fig. 5A). Surprisingly, this region also had low overall Shannon entropy, indicating that it was well-predicted *in vivo* and likely maintained a single conformation within cells. To understand this better, we performed ΔSHAPE analysis to measure the difference between *in vitro* SHAPE reactivity and *in vivo* SHAPE reactivity. Positive ΔSHAPE values usually indicate protection from modification in the cellular environment (suggestive of protein binding), and negative ΔSHAPE values suggest enhanced SHAPE reactivity in cell (suggesting more dynamic structure *in vivo*). We found that the region of poor correlation between *in vivo* and *in vitro* maps (8086 nt to 8286 nt) corresponds to a highly positive ΔSHAPE region (Fig. 5C). The *in vitro* structure predicted within this region was a small stem that contained two bulges (Fig. 5D). Each bulged substructure displayed low SHAPE reactivity (average, 0.15) *in vivo* compared with that *in vitro* (average = 0.618), suggesting that this region might contain a stable protein binding site (Fig. 5D).

### Global and local evolutionary analyses suggested a functional role for novel identified structures.

The *in vitro* and *in vivo* SHAPE-MaP experiments revealed a large collection of novel RNA structural elements, and given that these substructures are so numerous, it was important to create a strategy for prioritizing them for functional analysis. Combining *in vitro* and *in vivo* results, we identified the 20 most well-folded structures within the collection (Table 1) (Materials and Methods section “Identification of well-folded regions”). To determine whether these secondary structures are evolutionarily conserved, thereby implying a functional role, we performed a synonymous mutation rate (dS) analysis (34) to measure the evolutionary pressure on the HCV genome. Previous work has suggested double-stranded nucleotides have lower dS values than single-stranded nucleotides in viral RNAs, which reflects the evolutionary pressure to maintain base-pairing interactions (12, 35). This method has been used successfully to identify conserved structures in multiple viruses (23, 24, 36).

As a first step for conducting phylogenetic analysis, we created two multiple sequence alignments, as follows: (i) a pan-genotype alignment that includes 126 full-length HCV sequences from all 7 HCV genotypes and (ii) a genotype 2-specific alignment that includes 7 full-length HCV genotype 2 sequences. We then calculated dS using FUBAR (37), binning the dS based on strandedness, as determined in our *in vitro* model (Materials and Methods section “Synonymous mutation rate analysis”). A codon was considered to be double stranded or single stranded depending on whether or not the third position of the codon was base paired.

**FIG 6:**
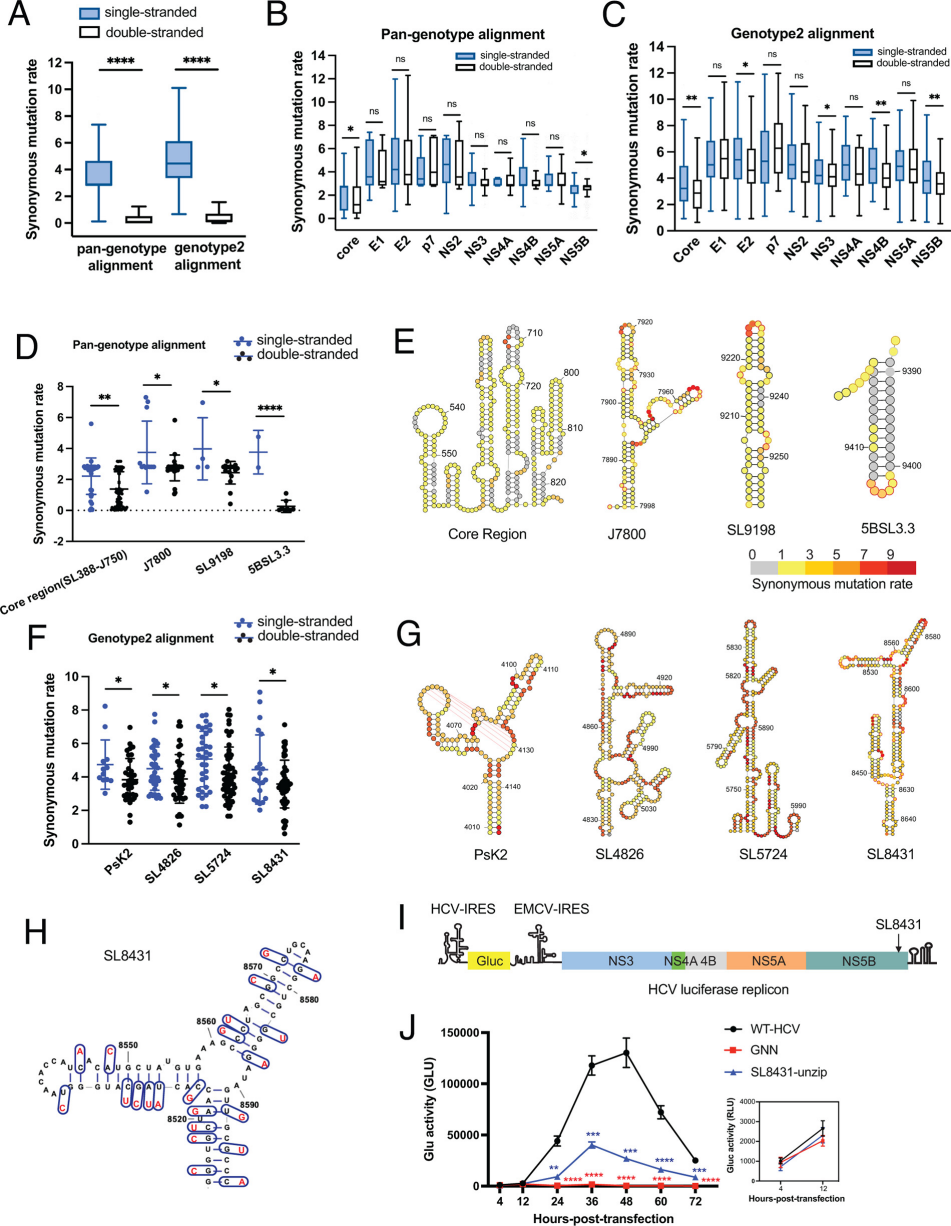
Global and local evolutionary analyses suggest a functional role for structures in the HCV ORF. (A) Synonymous mutation rates (dS) calculated for single- and double-stranded nucleotides across all genotypes and within genotype 2 by using the *in vitro* structural model. (B and C) A comparison of dS for single- and double-stranded nucleotides within individual HCV domains using the pan-genotype and genotype 2-specific alignments, respectively. (A to C) Data were plotted as in Fig. 1B. (D) Synonymous mutation rates calculated for four individually structured domains using the pan-genotype alignment. The line indicates median, and whiskers indicate standard deviation. (E) Four structures supported by dS analysis across all 7 genotypes, color-coded with dS. (F) Synonymous mutation rates calculated for four individual structured domains by using the genotype 2-specific alignment. Data are plotted as in panel D. (G) Four structures supported by dS analysis calculated by using the genotype 2-specific alignment, color-coded by dS. (H) Schematic showing the unzipped synonymous mutation for region SL8431. (I) Schematic of HCV luciferase replicon. Position of SL8431 is indicated. (J) Replication time courses for WT HCV, the replication-defective mutant (GNN), and unzip mutant of SL8431, measured by Gluc activity. Experiments were performed in triplicate; values were normalized to untransfected controls. Inset: Expanded plot for 4 h and 12 h posttransfection. **, *P* < 0.01; ***, *P* < 0.001; ****, *P* < 0.0001 by equal variance unpaired Student’s *t* test (mutant versus WT-HCV). Insignificant *t* test results (*P* > 0.05) are not displayed on the plot.

This analysis revealed a significantly lower dS at double-stranded regions for both types of alignments (*P* < 0.0001) (Fig. 6A). This result suggested that the overall structuredness (BPC of 60%) and strandedness we report for HCV genotype 2a were conserved in all 7 genotypes, indicating that an abundance of secondary structure is a general attribute of human hepaciviruses.

To determine whether certain sections of the HCV ORF experience particularly strong evolutionary pressure to maintain specific secondary structures, we applied dS analysis to individual protein-coding domains within the ORF. Expanding on previously published results (12), we found that regions encoding the viral core and NS5B genes have significantly lower dS at double-stranded regions, suggesting that these regions contain hubs of conserved RNA structure in all HCV genotypes (Fig. 6B). Intriguingly, when we performed a more restricted analysis using the genotype 2-specific alignment, we found that, in addition to the core and NS5B domains, the E2-, NS3-, and NS4B-coding regions also have significantly lower dS at double-stranded regions (Fig. 6C). This finding indicates that domain E2, NS3, and NS4B may contain genotype 2-specific structures that contribute to function in that subgroup of viruses.

To further examine evolutionary support for individual RNA substructures, we applied the dS analysis to all 20 well-folded regions that were identified *in vitro* and *in vivo*. Indeed, we found that several functional elements demonstrated previously to play important roles in the HCV life cycle were flagged as evolutionarily conserved in the pan-genotype alignment. Specifically, they included a well-folded region within the core-coding region (SL588, SL669, and J750) and three additional stems (J7800, SL9198, and 5BSL3.3) (Fig. 6D and E). These findings show that known riboregulatory regions appear as conserved and can be identified by using dS analysis. When the same analysis was performed by using the genotype 2 alignment, we identified the four regions described above, along with four novel structures (PsK2, SL4826, SL5724, and SL8431). These regions display significantly lower dS values in double-stranded regions (Fig. 6F and G), suggesting that they are conserved and functionally relevant in genotype 2 viruses. All four regions were highly structured (average BPC of 65%), with almost identical base-pair connectivities in both *in vivo* and *in vitro* structural models. Specifically, PsK2 was one of the novel pseudoknots we identified in the NS3-coding region. SL5724 and SL4826 were two long stems with a complex topology of internal loop structures. SL8431 consisted of several long stems and was particularly interesting because it contained a stable, multihelix junction (BPC of 77%).

Because of its unusually complex secondary structure and high level of conservation, we prioritized SL8431 for functional validation by using viral genetics. Specifically, we introduced mutations that disrupt the base-pairing interactions within the multihelix junction, without changing the identity of encoded amino acids (i.e., synonymous mutations) (Fig. 6H). In designing these mutants, we specifically avoided codons that were underrepresented in the wild-type virus. The effects of the resulting mutations on viral replication were then measured by using a well-characterized luciferase reporter assay (2), in which an HCV-SGR construct containing a Gaussia princeps luciferase (Gluc) reporter was inserted between the IRES structure and EMCV IRES structure, thereby providing a quantitative readout for the efficiency of viral replication (Fig. 6I). A lethal NS5B mutation (GNN), which disrupts the NS5B polymerase active site, was used as a control (38). Because the GNN construct cannot replicate, any luciferase activity detected from this construct represents background translation of the transfected RNA.

The resulting time course shows that HCV replication was severely impaired by mutants that unzip key helices within SL8431, resulting in an ∼5-fold defect at 48 h posttransfection (Fig. 6J). Importantly, there is no significant difference in luciferase activity between SL8431 and the GNN negative control at 12 h posttransfection, indicating that translation was not disrupted by the codon choices used to create the unzipped SL8431 mutant (Fig. 6J, inset). Additional structural compensatory mutants will ultimately need to be tested to determine if the defect comes exclusively from structural disruption. Taken together, these results suggest that SL8431 is important for HCV replication efficiency, and they further confirm the utility and efficacy of our pipeline for identifying potentially functional riboregulatory elements within long RNAs.

### Functional analysis of pseudoknots found within the HCV coding region.

**FIG 7:**
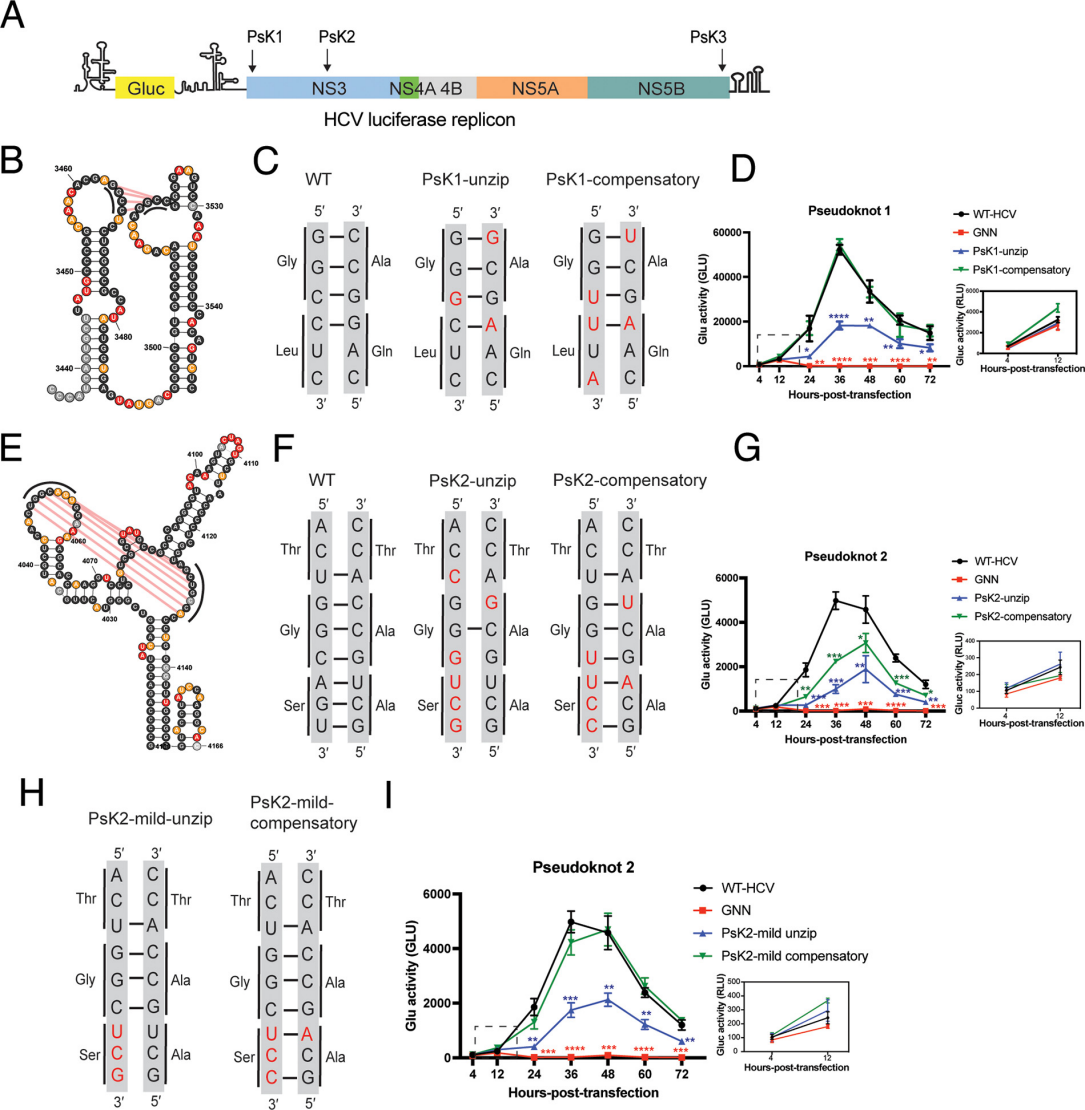
Mutational analysis supports a functional role for PsK1 and PsK2. (A) Schematic of the HCV Gluc replicon indicating the genomic positions of the three pseudoknots. (B and E) Structural representation of pseudoknot 1 (PsK1) and pseudoknot 2 (Psk2), labeled with *in vivo* SHAPE reactivity. Pseudoknotted nucleotides are indicated with black lines. Pseudoknot base pairs are labeled with red lines. (C, F, and H) Sequences of the pseudoknotted stems with unzipped and compensatory mutations. (D, G, and I) Replication time courses for WT HCV, the replication-defective mutant (GNN) control, and the unzipped and compensatory mutants of each indicated pseudoknot, measured by Gluc activity. Experiments were performed in triplicate, and values were normalized to untransfected controls. The same controls (WT HCV, GNN) are shown in panels G and I. Inset: Expanded plot for 4 and 12 h posttransfection. *, *P* < 0.05; **, *P* < 0.01; ****, *P* < 0.0001 by equal variance unpaired Student’s *t* test (mutant versus WT-HCV). Insignificant *t* test results (*P* > 0.05) are not displayed on the plot.

Our pseudoknot prediction pipeline revealed the presence of three novel pseudoknots in the HCV genome with potential regulatory function (PsK1, PsK2, and PsK3). Given the lack of knowledge about RNA tertiary structures in the ORF region, and the important role played by pseudoknots in the function of many different viruses, we decided to focus our functional analysis on the three novel pseudoknots. As described in the previous section, we introduced synonymous mutations to break or rescue the base pairs in the pseudoknot structures. The effects of these mutations on viral replication were then measured by using the luciferase reporter assay described above (Fig. 7A).

PsK1 was located within the NS3-encoding region and contained two small stem-loops, which were linked with four consecutive G-C pseudoknotted base pairs (Fig. 7B). Disruption and suppressor mutations for PsK1 were designed by unzipping three out of four base-pair interactions and then rescuing them by inserting G-U wobble and A-U base pairs, resulting in PsK1-unzip and PsK1-compensatory mutants, respectively (Fig. 7C). The resulting time course shows that unzipping PsK1 results in an ∼3-fold decrease in replication efficiency at 36 h posttransfection. Remarkably, the compensatory mutation completely restored efficient RNA replication, with no significant differences from the wild type at every time point collected (Fig. 7D). Importantly, both PsK1-unzip and PsK1-compensatory mutants have luciferase activity comparable to the replication-defective GNN mutant at 12 h posttransfection, indicating that PsK1 does not play a role in translation (Fig. 7D, inset), which differentiates it from the frame-shifting pseudoknots in other viruses (39, 40). Taken together, these results support the existence and importance of PsK1.

PsK2 was also located within the NS3-encoding region and consisted of a central loop and two neighboring stem-loops. The pseudoknotted region was composed of 7 consecutive base pairs between the central loop and the 5′ stem-loop (Fig. 7E and F, left). A PsK2 disruption mutant (PsK2-unzip) was designed to unzip 6 out of 7 base-pair interactions, which were then restored in a mutant containing five compensatory base pairs, including 2 G-U wobble base pairs (PsK2-compensatory) (Fig. 7F). The resulting time course shows that the PsK2-unzip mutant results in 3- to 5-fold decrease in HCV replication at 36 and 48 h posttransfection. In this case, the PsK2-compensatory mutant only partially rescued the RNA replication phenotype, retaining an ∼2-fold decrease in replication compared with the wild type (Fig. 7G). To examine whether the incorporation of G-U wobble base pairs caused the incomplete rescue, two additional mutants were designed in which only the last three base pairs at the base of the pseudoknotted stem were unzipped (PsK2-mild-unzip). The corresponding compensatory mutant (PsK2-mild-compensatory) incorporated only G-C and U-A corrective base pairs (Fig. 7H). To our surprise, we found that the PsK2-mild-unzip mutant exhibited replication defects comparable to PsK2-unzip, suggesting that the last three base pairs of this stem are the key elements for PsK2 function (Fig. 7I). Importantly, the replication curve of the PsK2-mild-compensatory mutant resembles that of the wild-type replicon, indicating that the PsK2-mild-compensatory mutations completely rescued RNA replication (Fig. 7I). The last three base pairs in PsK2 are immediately adjacent to a neighboring stem (SL4088), suggesting that these two duplexes may coaxially stack, thereby stabilizing the overall tertiary fold. Similar to PsK1, there is no significant difference between PsK2 mutants and the GNN control at 12 h posttransfection, demonstrating that PsK2 functions in HCV replication but does not impact translation (Fig. 7G and 7I, inset).

**FIG 8:**
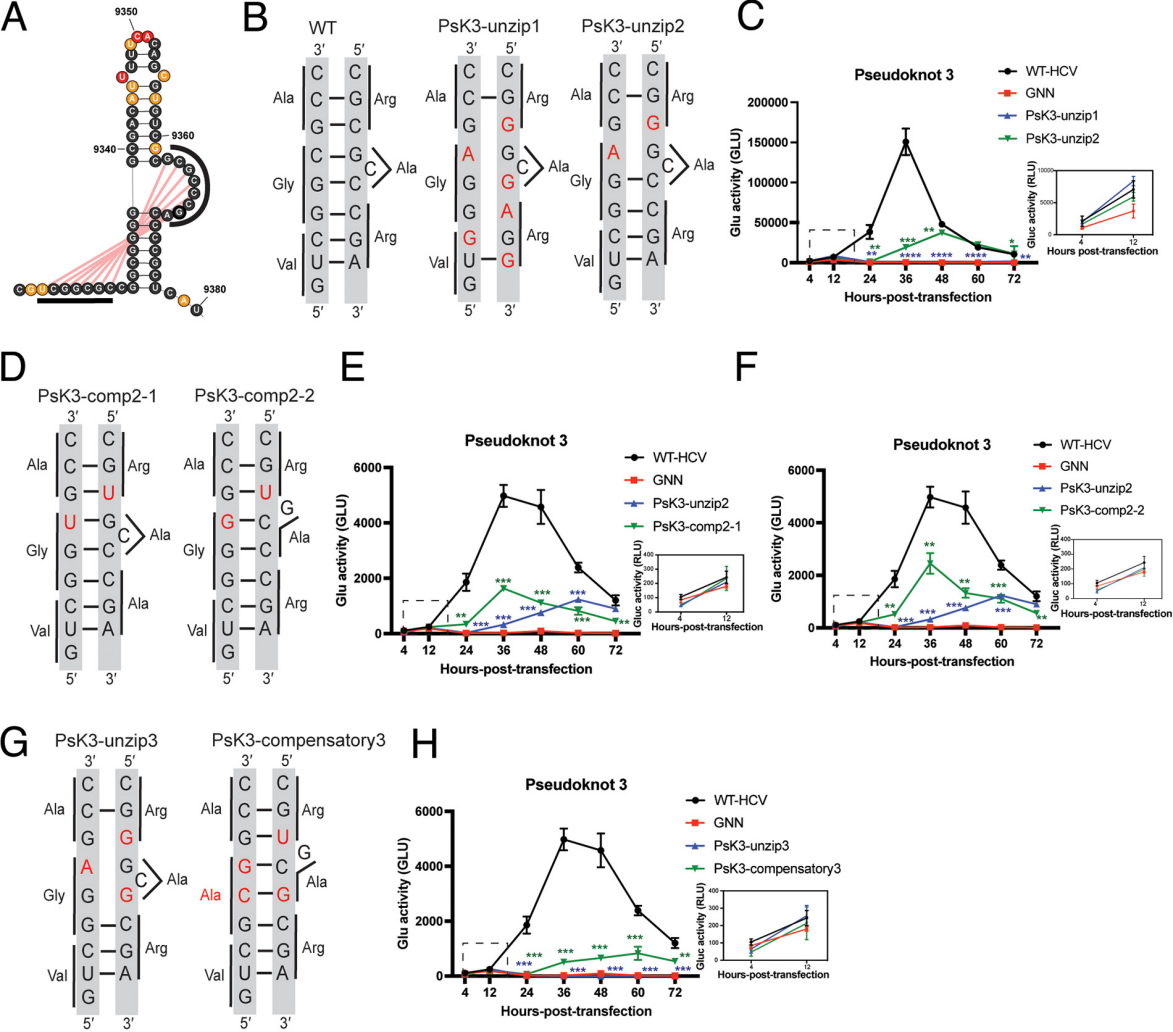
Mutational analysis supports a functional role for PsK3. (A) Structural representation of pseudoknot 3 (PsK3), labeled with *in vivo* SHAPE reactivity. Pseudoknotted nucleotides are indicated with black lines, and pseudoknot base pairs are indicated with red lines. (B, D, and G) Sequences for the unzipped and compensatory mutations in the pseudoknotted stem. (C, E, F, and H) Replication time courses for WT HCV, the replication-defective mutant (GNN), and the unzipped and compensatory mutants of PsK3, by Gluc activity. The experiment was performed in triplicate, with values normalized to untransfected controls. The same controls (WT HCV, GNN) are shown in panels E, F, and H. Inset: Expanded plot for the 4- and 12-h posttransfection time points. *, *P* < 0.05; **, *P* < 0.01; ***, *P* < 0.001; ****, *P* < 0.0001 by equal variance unpaired Student’s *t* test (mutant versus WT HCV). Insignificant *t* test results (*P* > 0.05) are not displayed on the plot. The *t* test results for GNN versus WT HCV are not displayed on the plot.

PsK3 was located at the 3′ terminus of the NS5B-coding region, with pseudoknot base pairs formed between nucleotides within the bulged stem and an upstream, single-stranded region (Fig. 8A). The bulged stem has been previously designated 5BSL3.2, and it is known to play a crucial role in the HCV life cycle (14, 15, 41). Previous reports have shown that this bulged loop forms at least two long-range interactions with other sections of the HCV genome and that disruption of these interactions completely abrogates viral replication (15, 33, 42, 43).

The initial PsK3 disruption mutant was designed to unzip 5 out of 7 bp (PsK3-unzip1) (Fig. 8B), and given the severity of these changes, it is perhaps unsurprising that this mutant did not replicate (Fig. 8C). To evaluate the functional importance of the PsK3 pseudoknot structure without disrupting either of the previously identified long-range interactions, we designed a second mutant (PsK-unzip2) that left the reported long-range interaction region (5′-GCCCGA-3′) intact (Fig. 8B). This particular mutant was designed to destabilize two of the seven pseudoknotted base pairs. Surprisingly, this mutant had low replication efficiency, but the start of replication was delayed (Fig. 8C). We then designed two compensatory mutants to rescue the replication of the PsK3-unzip2 mutant. PsK3-comp-2-1 incorporated only corrective G-U wobble base pairs, while PsK3-comp2-2 contained both G-U and G-C corrective base pairs (Fig. 8D). The resulting replication curves show that both compensatory mutants restore the onset of RNA replication. PsK3-comp2-2 also increased the replication efficiency relative to PsK3-unzip2 by approximately 2-fold, representing a partial rescue of the decreased replication efficiency phenotype (Fig. 8E and F).

Since the PsK3 conformation that we have inferred overlapped with other known pairing interactions (and all of these may in fact be important), we designed another set of unzip and compensatory mutants to specifically probe the PsK3 conformation (Fig. 8G). PsK3-unzip3 disrupted 3 out of 7 pseudoknotted base pairs, with 1 of the mutations located in a region known to form the long-range interaction (the mutated nucleotide is highlighted in bolded text: 5′-GC**C**CGA-3′ → 5′-GC**G**CGA-3′). We observed that PsK3-unzip3 lost the ability to replicate (Fig. 8H), which could be due to the disruption of PsK3, disruption of other putative interactions, or both. To determine whether PsK3’s function can be restored specifically, at least in part, we designed the PsK3-compensatory3 mutant, which restored the PsK3 conformation and included a single, unavoidable nonsynonymous mutation (Gly→Ala). To our surprise, despite the amino acid change, the PsK3-compensatory3 afforded a mild rescue of the severe replication defect, indicating that PsK3 conformation indeed plays at least one role in the conformational ensemble. The fact that PsK3-compensatory3 does not fully restore RNA replication suggests that other conformations are also essential for viral replication. The similar luciferase activity observed at 12 h posttransfection for all PsK3 mutants and the GNN control suggests that the disruption of PsK3 does not affect translation (Fig. 8C, E, F, and H, insets). Overall, these results show that unzipping of PsK3 results in severe replication defects and that compensatory mutations can rescue the phenotype only partially, suggesting that PsK3 exists within a complex interaction environment.

Taken together, our ability to identify novel, functional pseudoknots in the HCV ORF not only underscores the power of our prediction pipeline but also highlights the existence and importance of three novel predicted pseudoknots, of which all appear to be important for viral replication.

## DISCUSSION

### An abundance of functional RNA structural elements within the HCV genome.

In this work, we present the complete secondary structure of the HCV RNA genome, revealing a multitude of complex architectural units that are found in all sections of the genome, throughout the UTRs and long open reading frame. The resulting secondary structural maps revealed a network of riboregulatory elements with the potential to modulate diverse aspects of the viral life cycle. These findings expand and build upon earlier mechanistic studies showing that specific long-range interactions within the HCV genome are critical for the regulation of replication and infectivity (33, 43–45). Here, we show that the motifs discovered earlier are not isolated cases but are members of a vast set of architectural elements that span the entire genome.

An analysis of the HCV RNA secondary structure map revealed a number of interesting structure targets. To rank and prioritize them for functional validation, we employed the well-established “low Shannon, low SHAPE” (lowShannonShape) metric, which indicates regions that are not only thermodynamically stable but also likely to maintain a single conformation (27). By using this metric, we identified 20 well-folded structures, including some long-stem structures (e.g., SL2140, SL3020, SL6038, SL7500, and SL8431) and some highly compact short-stem structures (core regions, PsK2, SL5087, and PsK3) (Table 1). Among them, SL8431 has the highest BPC and lowest average SHAPE reactivity, and thus, we prioritized this structure for functional validation. Indeed, we found that unzipping SL8431 resulted in a 5-fold decrease in HCV replication efficiency. Since most HCV replicase proteins function in *cis* (46), this RNA region may contain a replicase protein binding site that recruits the HCV replication complex.

A comparative analysis of SHAPE-MaP data *in vitro* versus *in vivo* (ΔSHAPE) suggested the presence of specific protein-binding sites in the viral genome (47). For example, the loop region of SL8145 had significantly lower SHAPE reactivity *in vivo* than that *in vitro*. Indeed, published CLIP data sets indicate that the m6A reader protein YTHDF2 interacts with SL8145 (48). SL8145 contains a consensus m6A modification motif (DRAmCH, D = G/A/U, R = G > A, and H = U/C/A), raising the possibility that SL8145 may be m6A modified and that this complex with YTHDF2 serves some pro- or antiviral function.

### Pseudoknots within the HCV genome.

Pseudoknots are among the most prevalent RNA tertiary structures. They can adopt stable three-dimensional (3D) structures that contribute to diverse functions in viral biology (16). For example, the xrRNA1 pseudoknot in the dengue virus 3′ UTR is important for stalling the Xrn1 exonuclease, thereby preventing RNA degradation (49, 50). Furthermore, a pseudoknot in the HCV 5′ UTR IRES directs the binding of the ribosomal 40S subunit to the start codon (51). However, the majority of functional pseudoknots in viral ORF regions described to date contribute to programmed ribosomal frameshifting, such as the frameshifting pseudoknots in HIV, a subset of flaviviruses, and all coronaviruses (52–54). Here, we report three novel pseudoknots in the HCV ORF that impact viral replication rather than translational frame shifting, thereby broadening the functional repertoire of viral pseudoknots.

PsK1 and PsK2 are located in the NS3 coding region. Previous work has demonstrated that NS3 helicase activity is required in *cis* for HCV replication (46), suggesting a potential protein-RNA interaction at the NS3 locus. PsK1 and PsK2 maintain highly compact structures *in vivo* and their genetic disruption decreases HCV replication efficiency by 3- to 5-fold. These pseudoknots may serve as binding sites for HCV viral proteins, perhaps recruiting the components of the replication complex.

PsK3 lies within a *cis*-regulatory element (CRE) at the 3′ end of the NS5B ORF (14, 15). The bulged stem-loop in PsK3, also known as 5BSL3.2, is an unusually compact region of the genome. 5BSL3.2 is involved in long-range interactions with the 5′ and 3′ ends of the genome, the protein NS5B, and the ribosomal 40S subunit (14, 41, 42, 45). Since these interactions are mutually exclusive, this region has been proposed to function as a switch that toggles between conformations to regulate both viral replication and translation (3, 42). Our study has elucidated the short-range PsK3 interaction and its role in regulating HCV replication. PsK3-disrupted mutants show a decrease in replication efficiency and a delay in replication initiation, suggesting that this pseudoknot is a transient conformation that contributes to the appropriate timing of viral replication.

### The HCV genome structure is maintained in human cells.

One of the most significant aspects of this study is our finding that structural features within the HCV genome were largely independent of the environmental context; similar structural elements were observed for HCV RNA probed *in vivo* versus after *in vitro* transcription and purification. This finding is extremely important because there are very few studies in which the secondary structure of a large RNA molecule has been independently determined *in vitro* and *in vivo* (31, 55, 56). The two-tier design of this study provided us with the opportunity to test the frequently stated notion that RNA ORFs are always unstructured within eukaryotic cells, ostensibly because of an abundance of RNA binding proteins and translocating ribosomes (28–31). RNA viruses are ideal systems for addressing this issue because they are long mRNAs that are subjected to similar posttranscriptional and translational processes as those experienced by host mRNA in the cytosol. The fact that the HCV genomic RNA maintained most of the same structures *in vivo* as *in vitro* suggests that it contains persistent structural motifs that are not eliminated by the action of cytosolic components. This information indicates that highly stable RNA structural elements can persist (or refold) in the cellular environment and that one can build artificial genes in which secondary structures will persist, which has important implications for the design of mRNA vaccines and RNA devices in the cell.

### Stable cellular RNA secondary structure is directed by sequence.

The reported lack of secondary structure in eukaryotic and bacterial ORFs *in vivo* may be attributable to a lack of encoded secondary structure within these particular genes, which may not have been subjected to selective pressure to evolve a stable architecture. By design, our study contained an internal control that enabled us to directly test this idea; inserted within the ORF for the HCV replicon was a bacterial neomycin resistance gene and its structure was probed alongside the immediately adjacent viral genes. We observed that the neomycin ORF is almost devoid of secondary structure, while right next to it, the HCV genes adopted their typical elaborate architectures. Both segments of this RNA were exposed to the same action of ribosomes and other cytosolic factors, and yet only the HCV sequences folded. This result indicates that UTRs and ORFs can adopt discrete folded structures *in vivo* but only if this capability is encoded within the sequence. The HCV ORF has been selected to adopt a structured, folded architecture that persists in the cell, and it is an inherent attribute of its specific sequence, as originally hypothesized by Simmons (7). Interestingly, there are other viral genomes, particularly flaviviruses, that have been probed within infected cells, and they show a lower secondary structural content than we reported here for HCV (24, 31). Indeed, this result was also predicted by Simmons, based on comparative calculations of global secondary structure stability and conservation for hepacivirus and flavivirus genomes (7). Taken together, our results suggest that HCV (like betacoronaviruses) (22, 23) has an encoded propensity to adopt specific, highly folded RNA substructures *in vivo* and that it plays some role in the viral life cycle or during interplay with the host innate immune system.

### Correlation between secondary structure stability and features of viral genome organization.

Local trends in secondary structure propensity (or base pair content [BPC]) can be used to ascertain trends with potential relevance to function. An analysis of SHAPE-MaP data across the HCV genome revealed regions of various structural complexities. For example, segments encoding NS2, NS4B, NS5A, and the second half of NS5B have a high BPC (>60%), while regions encoding E2 and the first half of NS5B are almost single stranded (BPC of <25%).

Previous studies on viral RNAs have shown that high BPC structures often serve as hubs for protein-binding sites (e.g., HCV CRE, and 3′ UTR), while low BPC regions help maintain overall flexibility of the RNA (14, 44). In the NS5B-coding region, we identified several regions with low BPC (8875 to 9062 and 9378 and9431) adjacent to previously identified high BPC structures involved in important long-range interactions (SL9074 and 5BSL3.2) (33, 43). We speculate that the unstructured regions in NS5B may facilitate the formation of the rigidified long-range interactions and that unstructured regions provide sufficient flexibility for dynamic structural sampling. It is likely that high BPC and low BPC regions function synergistically for efficient structural regulation, suggesting the importance of investigating both highly structured elements and the flanking unstructured regions to understand the role of two-dimensional (2D) and 3D genome architecture in genomic folding. The structural compartmentalization we report here is reminiscent of DNA chromatin architecture. Long RNA genomes may also organize their genomes into discrete 3D-functional units, much like the topological associating domains found in the chromatin of eukaryotes (57). It will be interesting to conduct additional cross-linking experiments on RNA genomes to further explore this hypothesis.

### HCV and SARS-CoV-2 RNA use different structural strategies for genome organization.

The same probing and prediction methods employed in this study were also applied to another RNA virus, namely, severe acute respiratory syndrome coronavirus 2 (SARS-CoV-2) (23), providing an opportunity for a comparative study of the two viruses. Despite comparable levels of overall base pair content (60%), we noticed that SARS-CoV-2 and HCV have different strategies for arranging their secondary structures. SARS-CoV-2 has many short, locally folded stem-loops, while HCV contains relatively long stem-loops (e.g., SL2140, SL3020, SL4200, and SL6038). Additionally, the multihelix junctions in the SARS-CoV-2 genome tend to be composed of many short stem-loops radiating from a central junction (e.g., region 15 from Huston et al. [23]), while the multihelix junctions in HCV tend to contain small central loops with fewer branching stems (e.g., SL8431).

Both strategies may play specific roles in evading the innate immune system. The short stems in SARS-CoV-2 may help the virus escape from pattern recognition receptors, such as MDA5, which recognizes long RNA duplexes (58). In contrast, the long stems and stable multihelix junctions in HCV may bring together distant parts of the viral sequence, resulting in a compact, globular genome architecture that may protect the viral genomic RNA from pattern recognition receptors and cellular nucleases (7, 59).

The distinct genome architectures of these two viruses indicate that they face different selective pressures and that, in response, they have developed different strategies for organizing their secondary structures. Although it is well accepted that a highly structured genome may provide protection from the innate immune system and an enhancement of RNA stability (60), different RNA viruses may have adapted to different constraints, resulting in a secondary structural organization that is much like an RNA fingerprint. Understanding the genomic viral RNA structure may therefore enhance our mechanistic understanding of viral evolution and fitness.

### Concluding remarks.

In this study, we provided a full-length structural model of the HCV RNA genome and showed that it maintains a highly compact structure *in vivo*. We established the functional importance of individual RNA structural elements through evolutionary and genetic studies, identified a novel role for pseudoknots in viral ORFs, and analyzed correlations between architectural complexity and genomic context. Together, these data provide a functional atlas of the HCV genome structure and lay the foundation for additional mechanistic and pharmacological studies. Importantly, the significance of the study extends beyond its implications for molecular virology. The analysis pipeline can be applied to other studies of long RNA transcripts, such as long noncoding RNAs, and the study provides much-needed comparative data on the differences between RNA structure in the cell and RNA that has been purified and studied *in vitro*. The implications of the study therefore have broad implications for RNA science.

## MATERIALS AND METHODS

### HCV RNA *in vitro* transcription, semi-native purification, folding, and SHAPE modification.

The *in vitro* transcription and semi-native purification of the full-length HCV RNA genome (Jc1, sequence available at GitHub repository) (61) were performed as described previously (17). Briefly, the RNA was *in vitro* transcribed by runoff transcription using T7 RNA polymerase P266L variant (62) and treated with DNase (Promega) and proteinase K (ThermoFisher). Then, 25 mM EDTA (pH 8.0) was added to chelate Mg^2+^, and the RNA was concentrated by using 100-kDa Amicon Ultra filtration columns at room temperature. Size-exclusion chromatography was performed at room temperature by using a self-packed Sephacryl S-1000 column with a 24-mL bed volume and equilibrated with filtration buffer (50 mM K-HEPES [pH 7.5], 150 mM KCl, and 100 μM EDTA). RNA from the peak fraction was diluted to 250 ng/μL and was folded by incubating with 10 mM MgCl_2_ at 37°C for 30 min. A final concentration of 200 mM NAI or the same volume of DMSO was added to the RNA solution and incubated for 10 min at 37°C. RNA was then LiCl-precipitated, washed with 80% EtOH, and resuspended in RNA storage buffer (10 mM morpholinepropanesulfonic acid [MOPS; pH 6.0] and 1 mM EDTA).

### Reverse transcription and library preparation for *in vitro* SHAPE-MaP.

*In vitro*-purified RNA (1 μg) was reverse transcribed by using 200 U SuperScript II under standard buffer conditions (50 mM Tris-HCl [pH 8.3], 75 mM KCl, 10 mM dithiothreitol [DTT], 6 mM MnCl_2_, and 0.5 mM deoxynucleoside triphosphate [dNTP] mix) with random hexamers (LifeTech). Second strand synthesis was performed by using NEBNext Ultra II nondirectional second strand synthesis module (New England BioLabs [NEB]). Double-stranded cDNA was purified with Monarch DNA cleanup kits (NEB) and diluted to 0.2 ng/μL. Sequencing libraries were generated by using a Nextera XT DNA library preparation kit (Illumina) according to the manufacturer’s instructions. Libraries were quantified by using a Qubit instrument (Life Technologies) and bioanalyzer (Agilent) and sequenced on a NextSeq 500/550 system (Illumina).

### Cell collection, in-cell modification, reverse transcription (RT), and library preparation.

To perform *in vivo* SHAPE-MaP, Huh-7.5 cells constitutively expressing the HCV subgenomic replicon (SGR-neo-JFH-1) (26) were grown to 90% confluence. The cells were washed with ice-cold phosphate-buffered saline (PBS), collected by cell scraper, centrifuged at 1,000 × *g* for 5 min, and then resuspended in ice-cold PBS. A final concentration of 200 mM NAI or the same amount of DMSO was added to the cells. The reaction mixture was incubated at room temperature for 10 min. RNA was extracted by the addition of TRIzol according to manufacturer’s recommendations and then purified with the Qiagen RNeasy kit. RNA was then resuspended in RNA storage buffer to a final concentration of 1 μg/μL. RNA (1 μg) was reverse transcribed with SuperScript II by using gene-specific primers. Following reverse transcription, the cDNA products were cleaned up by using Agencourt AMPure XP beads and amplified by using Q5 hot start polymerase (NEB). RT primers and PCR primers for the amplicons are available at the GitHub repository.

### SHAPE-MaP data analysis.

All SHAPE-MaP libraries were analyzed by using ShapeMapper 2 (63). The *in vitro* SHAPE-MaP library was aligned to the sequence of HCV Jc1 (based on JF343782.1). The raw sequencing files (.fastq files) for the *in vivo* SHAPE-MaP were concatenated first and then mapped to HCV pSGR-JFH1 (based on AB114136.1). The sequence files of the HCV genome used in the SHAPE-MaP analysis are available at the GitHub repository. All libraries passed the three quality-control checks of ShapeMapper 2.

### Pseudoknot prediction and secondary structure modeling.

ShapeKnots (implemented in RNAstructure v5.8 [20]) was used to identify pseudoknots in the HCV genome. *In vitro* SHAPE reactivity replicate 1 data were used as a soft constraint with default slope and intercept parameters (slope, 1.8; intercept, −0.6). Secondary structure (with pseudoknots) was predicted in 500-nt sliding windows with a 100-nt step size across the HCV genome. Using the well-characterized IRES-pseudoknot as a benchmark (11), we set up the following criteria for identifying each new pseudoknot: (i) the pseudoknot was observed in two adjacent windows in the lowest energy structure prediction, and (ii) nucleotides predicted to be involved in the pseudoknot helix had low SHAPE reactivities. These criteria successfully predicted the HCV IRES pseudoknot with 100% positive predictive value (PPV) and sensitivity.

The full-length structural model was predicted using Superfold, which performs a partition function calculation and a minimum free energy calculation in sliding windows. This process generates a consensus structural model for long RNAs (21). The *in vitro* or *in vivo* NAI SHAPE reactivities from replicate 1 were used as constraints using default slope and intercept parameters. The maximum base-pair distance was set to 500 nucleotides, as done in the previous studies (23). The predicted pseudoknot coordinates were included as hard constraints in the Superfold secondary structure prediction.

Structure prediction accuracy was calculated using Scorer (implemented in RNAstructure v5.6 [64]). Scorer calculates the positive predictive value (PPV) by comparing the HCV IRES structure prediction with the published crystal structure (equation 1) (11).

(1) $$\text{PPV = }\frac{\text{number of correctly predicted base pairs }}{\text{total number of predicted base pairs}}\times 100\%$$

All structures and reactivities shown in this manuscript were drawn using StructureEditor (64). The full-length structure (.ct file) and reactivities from both *in vitro* model and *in vivo* model are available at the GitHub repository.

### Local base pair content (BPC) analysis.

BPC is a metric describing overall RNA secondary structural complexity, computed by calculating the percentage of double-strand character within a given transcript (22). In this manuscript, we analyzed local base pair content by calculating BPC using a 50-nt sliding window across the HCV genome in steps of 1 nt. Local BPC values were plotted in a heatmap in Fig. 2.

### Identification of well-folded regions (low-Shannon, low-SHAPE).

Shannon entropy for each nucleotide was derived from the Superfold output. The local median SHAPE reactivity and Shannon entropy values were calculated using a centered 50-nt sliding window (25 nt flanked on each side). Global median SHAPE reactivities and Shannon entropies were subtracted from the calculated local medians for the purpose of identifying low Shannon entropy and low SHAPE reactivity regions. Regions of 40 nucleotides or longer with local median SHAPE reactivities and Shannon entropies below the global median are considered well-folded. In certain cases, identified well-folded regions were expanded to include the complete structure (SL4826 and J7800). The complete collection of well-folded regions identified from *in vitro* and *in vivo* data sets is shown in Table 1.

### Delta SHAPE analysis.

Delta SHAPE analysis was performed using the delatSHAPE program (47). The .map file output from *in vivo* and *in vitro* SHAPE analysis were used as the input. The region from 7877 to 8412 (HCV, Jc1) was included in the analysis. The standard deviation and Z-score threshold were set to default. The find cite threshold was set to 10,15. Smoothing window size was set to 10.

### HCV *Gaussia* luciferase activity assay.

Synonymous mutations in the ORF region containing pseudoknots were incorporated using the Q5 site-directed mutagenesis kit (NEB). The template plasmid was linearized and *in vitro* transcribed as described above and then purified by the Qiagen RNeasy kit. Then, RNA was resuspended in RNA storage buffer to a final concentration of 1 μg/μL. The luciferase activity assay was performed in 12-well plate format. HCV RNA was transfected with the MIR-2250 mRNA lipid transfection reagent (Mirus) according to the manufacturer’s protocol. At each time point, the medium was collected, cells were washed three times with phosphate-buffered saline, and fresh medium was added. The collected medium was clarified by centrifugation (5 min at 20,000 × *g*), placed into fresh tubes, mixed with one volume of 2× luciferase lysis buffer, and stored frozen(–80°C). Luminescence was measured on a Biotek Synergy H1 plate reader using 20 μL of the medium, 50 μL of *Gaussia* luciferase reagent (Pierce *Gaussia* luciferase flash assay kit; ThermoFisher), and a 5-s integration time.

### Synonymous mutation rate analysis.

The pan-genotype alignment was generated using 126 full-length HCV sequences from 7 genotypes. The genotype 2-specific alignment was generated using 7 full-length HCV genotype 2 sequences. The multiple sequence alignment (MSA) was generated using Clustal W codon alignment (implemented in MEGA7 [65]). Sequences with more than 95% similarities were removed. The synonymous mutation rate at individual codon sites was estimated using the phylogenetic-based parametric maximum likelihood FUBAR method (37). Each codon was categorized as being either a base paired or an unpaired codon depending on whether the third position nucleotide was paired in the *in vitro* structure model (24). The MSAs are available at the GitHub repository.

### Data availability.

Sequence files, primer data, SHAPE-MaP outputs (.map files), structural prediction outputs (.ct files), multiple sequence alignment files, and high-resolution structural map figures are available at the GitHub repository (https://github.com/pylelab/HCV_SHAPE_MaP_Structure).
